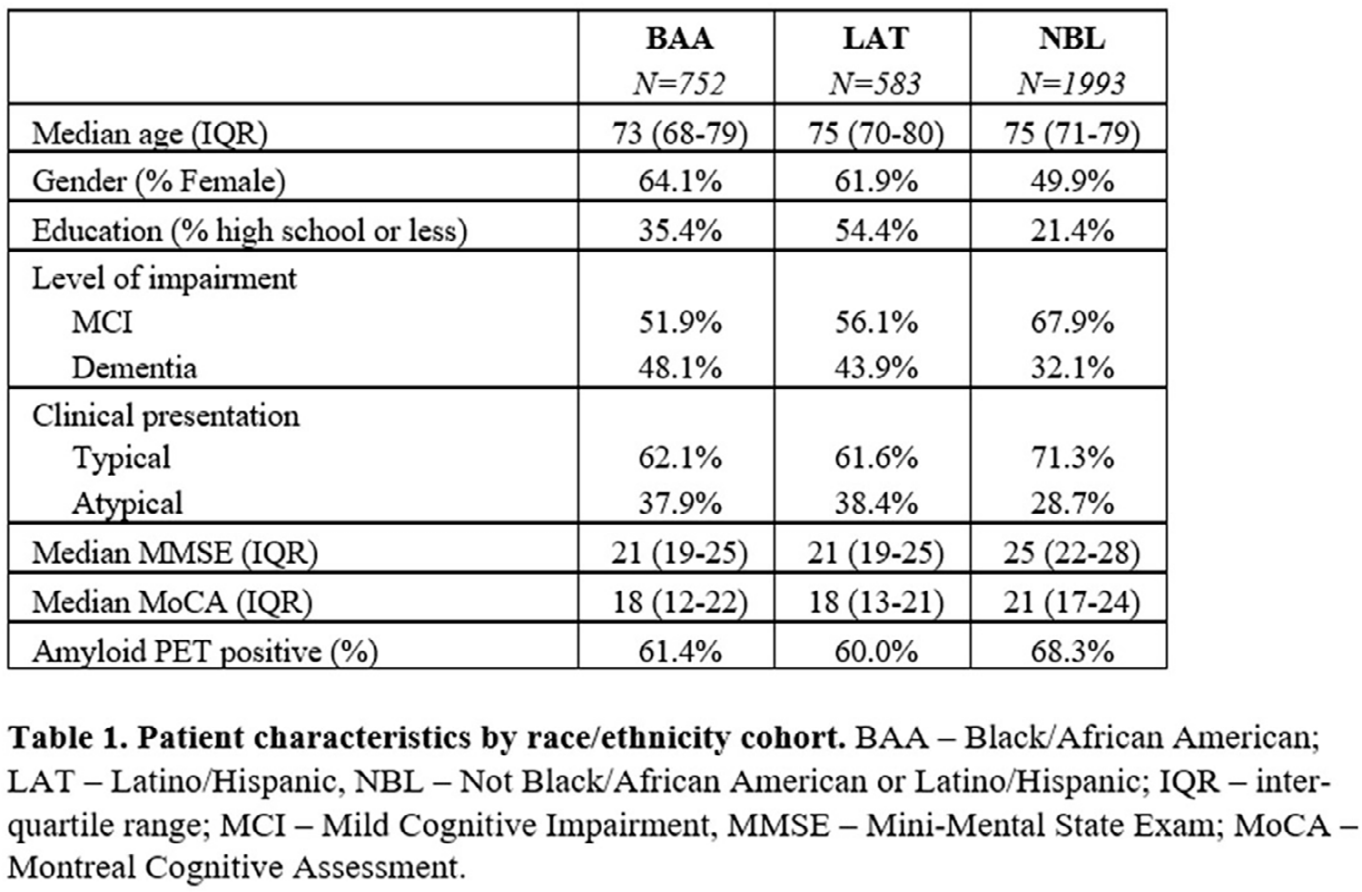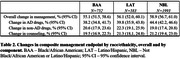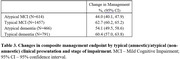# Clinical Impact of Amyloid PET in Diverse Medicare Beneficiaries with Cognitive Impairment: Preliminary Results from New IDEAS

**DOI:** 10.1002/alz.092654

**Published:** 2025-01-09

**Authors:** Gil D. Rabinovici, Constantine Gatsonis, Peggye Dilworth‐Anderson, Ilana F Gareen, Emily Glavin, Lucy Hanna, Bruce E Hillner, Andrew March, Justin Romanoff, Barry A Siegel, Karen Smith, Christopher J. Weber, Charles Windon, Rachel A. Whitmer, Consuelo H. Wilkins, Maria C. Carrillo

**Affiliations:** ^1^ Department of Radiology and Biomedical Imaging, University of California, San Francisco, San Francisco, CA USA; ^2^ UCSF Alzheimer's Disease Research Center, San Francisco, CA USA; ^3^ Memory and Aging Center, Weill Institute for Neurosciences, University of California, San Francisco (UCSF), San Francisco, CA USA; ^4^ Brown University, Providence, RI USA; ^5^ University of North Carolina, Chapel Hill, Chapel Hill, NC USA; ^6^ Dept. of Biostatistics, Brown University, Providence, RI USA; ^7^ American College of Radiology, Reston, VA USA; ^8^ Virginia Commonwealth University, Richmond, VA USA; ^9^ Washington University in St Louis, St. Louis, MO USA; ^10^ Memory and Aging Center, UCSF Weill Institute for Neurosciences, University of California, San Francisco, San Francisco, CA USA; ^11^ Alzheimer's Association, Chicago, IL USA; ^12^ Memory and Aging Center, Weill Institute for Neurosciences, University of California, San Francisco, San Francisco, CA USA; ^13^ Department of Neurology, University of California, San Francisco, San Francisco, CA USA; ^14^ University of California, Davis, Davis, CA USA; ^15^ Meharry‐Vanderbilt Alliance, Nashville, TN USA; ^16^ Vanderbilt University Medical Center, Nashville, TN USA

## Abstract

**Background:**

The Imaging Dementia—Evidence for Amyloid Scanning (IDEAS) study demonstrated that amyloid PET changes patient management in >60% of Medicare beneficiaries with MCI/atypical dementia. IDEAS had limited racial/ethnic diversity and excluded patients with “typical” amnestic clinical presentations. Here we present preliminary results from the New IDEAS study, which evaluates the clinical impact of amyloid PET in a more racially, ethnically and clinically diverse cohort.

**Method:**

Launched in December 2020, the New IDEAS study (NCT04426539) is recruiting Medicare beneficiaries with typical (amnestic) and atypical (non‐amnestic) MCI/dementia at 142 dementia specialty clinics across the U.S. Multifaceted, community‐engaged and culturally‐tailored strategies are implemented to enhance the diversity of the cohort. Based on self‐identified race/ethnicity, patients are enrolled into 1 of 3 cohorts: Black/African‐American (BAA), Latino/Hispanic (LAT), or not BAA or LAT (NBL, all other racial/ethnic identities). Patients undergo amyloid PET using an FDA‐approved tracer with local visual read at 127 imaging facilities. Changes in management are measured between the pre‐PET visit (intended management, assuming no access to amyloid PET) and 90‐day post‐PET visit (implemented management, incorporating amyloid PET results). A composite management endpoint captures changes in one or more of the following: AD drug therapy, non‐AD drug therapy, counseling about safety and future planning.

**Result:**

Out of 5,209 registered participants, 3,328 (63.9%) participants completed amyloid PET, pre‐ and post‐PET visits at time of analysis (median age 74; 55.2% female; 22.6% BAA/17.5% LAT). Demographic and clinical features by cohort are shown in Table 1. Compared to NBL, BAA and LAT cohorts presented with greater clinical impairment, more frequent atypical clinical presentations and lower rates of amyloid PET positivity. Changes in the composite management endpoint occurred in 57.5% of all participants (57.2% MCI, 58.1% dementia), with similar rates across cohorts (Table 2). Following PET, anti‐amyloid antibodies were newly recommended in 7.0% of BAA, 7.4% of LAT and 9.0% of NBL. Changes in management were more frequent in typical than atypical clinical presentations (Table 3).

**Conclusion:**

These preliminary findings highlight the clinical utility of amyloid PET in a diverse and representative sample of cognitively impaired patients recruited in real‐world practice.